# Generation of mAbs to foot–and–mouth disease virus serotype A and application in a competitive ELISA for serodiagnosis

**DOI:** 10.1186/s12985-016-0650-z

**Published:** 2016-11-28

**Authors:** Ming Yang, Wanhong Xu, Hilary Bittner, Jacquelyn Horsington, Wilna Vosloo, Melissa Goolia, Diana Lusansky, Charles Nfon

**Affiliations:** 1 National Centre for Foreign Animal Disease, 1015 Arlington Street, Winnipeg, MB R3E 3M4 Canada; 2 Australian Animal Health Laboratory, CSIRO, 5 Portarlington Road, Geelong, VIC 3220 Australia

**Keywords:** Foot–and–mouth disease virus, Serotype A, Monoclonal antibody, Serodiagnosis, Competitive ELISA

## Abstract

**Background:**

Foot–and–mouth disease (FMD) is an economically devastating disease that severely limits international trade of animals. Of the seven FMD virus (FMDV) serotypes, serotype A is one of the most widespread cross the world. Currently antibodies to FMDV are detected in animals using the virus neutralization test (VNT) and the enzyme-linked immunosorbent assay (ELISA). The VNT is laborious, time–consuming and reliant on live virus and cell cultures, while ELISA has the advantage of using inactivated antigens and often provides more reproducible results. The aim of this study was to develop a reliable and rapid competitive ELISA (cELISA) for the detection of antibodies to FMDV serotype A (FMDV/A).

**Results:**

A panel of FMDV/A specific monoclonal antibodies (mAbs) was generated and their ability to compete with a polyclonal serum from FMDV/A–infected cattle was examined. Two mAbs inhibited the binding of a polyclonal serum to FMDV/A viruses. The binding epitopes of each were determined as conformational and located on the VP2 viral capsid protein. The FMDV/A cELISA was developed using these two mAbs and FMDV/A inactivated virus as antigen. The diagnostic specificity and sensitivity were 99.7 and 99.3% (98.5–100%) respectively, based on a predetermined cut–off of 50% inhibition. When analysing sera from animals experimentally infected with FMDV/A, the cELISA detected antibodies from 5-days post infection (dpi) and remained positive for at least 21–28 days post infection. Comparison based on the Kappa coefficient showed strong agreement (90–94%) between cELISA and VNT.

**Conclusion:**

The cELISA results are comparable to the VNT for antibody detection making it a simple and reliable test to detect antibodies against FMDV/A.

**Electronic supplementary material:**

The online version of this article (doi:10.1186/s12985-016-0650-z) contains supplementary material, which is available to authorized users.

## Background

Foot–and–mouth disease (FMD) is one of the most highly contagious animal diseases with a broad host range, including cattle, buffaloes, pigs, sheep, goats and around 70 wildlife species. It is an economically devastating disease that severely constrains international trade of animals. The FMD virus (FMDV) is a non–enveloped virus containing a single–stranded RNA genome surrounded by an icosahedral capsid. The capsid comprises 60 copies each of four structural proteins: VP1, VP2, VP3 and VP4 [1]. There are seven serotypes of FMDV namely O, A, Asia 1, C, Southern African Territories (SAT) 1, SAT 2 and SAT 3. Outbreaks due to FMDV serotype A (FMDV/A) have been reported in all continents, including South Eastern Asia, Southern Asia, South America, Middle East, Eastern and Western Africa [2]. Within FMDV/A, strains are both antigenically and genetically diverse [3, 4]. A total of 26 regional genotypes within three continental topotypes have been reported for FMDV/A [5, 6].

Serological tests of FMDV are used for certification of animals prior to import and export, confirmation of FMDV infection and demonstration of vaccine efficacy [7]. One of the international standard tests for FMDV antibody detections is the virus neutralisation test (VNT). However, the VNT is costly and labour intensive rendering large scale serological testing difficult. In addition, the procedure requires live virus, thus confining the test to biocontainment level 3 laboratories in non–endemic countries. Competitive ELISAs (cELISAs) are commonly used for antibody detection due to their sensitivity, simplicity, ease to scale up to accommodate the screening of large numbers of serum samples and suitability to detect antibodies from different species without needing species–specific secondary antibodies [8, 9]. The use of mAbs in these tests provides a consistent supply of reagents that unlike polyclonal antisera, does not require the use of virus once mAbs have been generated. Several cELISAs to detect antibodies against FMDV non–structural protein (NSP) have been developed, validated and used in serological surveillance [9–13]. However, NSP ELISAs are serotype–independent and cannot be used in testing vaccine potency or monitoring the effectiveness of vaccinations. cELISAs can also be used post outbreak surveillance due to their specificity.

Ko et al. [14] reported a cELISA for anti-FMDV/A antibody detection using a monoclonal antibody (mAb) that was raised by immunization of mice with a VP1 peptide corresponding to the GH loop. The mAb used in that study demonstrated reactivity to two FMDV/A isolates (A24 Cruzeito/Brazil/55 and A22 Iraq) [14], but its reactivity to other strains, especially recent isolates, has not been examined. Furthermore, since the mAb was raised against a region that is known for its high variability, there is a possibility that the cELISA may not detect antibodies against all FMDV/A isolates.

The goal of this study was to generate mAbs and develop a simple and rapid serodiagnostic assay for FMDV/A, suitable for testing sera from all susceptible species exposed to a range of FMDV/A isolates. A FMDV/A cELISA based on two mAbs that recognize a surface antigenic site was developed and validated using serum samples from experimentally infected animals. The cELISA is suitable for use in diagnosis of FMDV/A infection, monitoring the effectiveness of field vaccinations, and epidemiological studies of FMD in animal populations.

## Results

### Production and characterization of mAbs against FMDV/A

Spleen cells from mice inoculated with BEI–inactivated FMDV/A22 Iraq 24/64 (A22 Iraq) were fused with myeloma cells. Twelve hybridomas specific for FMDV/A were generated from three fusions. After subcloning, the ability of the mAbs to compete with a polyclonal serum from cattle infected with FMDV/A was examined. Two mAbs (#5 and #7) clearly inhibited the binding of the polyclonal antibodies to FMDV/A. These two mAbs were characterized as IgG2a/*k* and IgG1/*k* respectively. The reactivity and specificity of the mAbs against different FMDV serotypes and other viruses causing vesicular disease were tested using a double antibody sandwich (DAS) ELISA [15]. Results indicated that mAb #7 was FMDV/A specific without cross–reactivity to other serotypes of FMDV or other vesicular disease viruses (Additional file 1: Table S1). However, mAb #5 showed cross–reactivity to four FMDV/C isolates (data not shown).

**Fig. 1:**
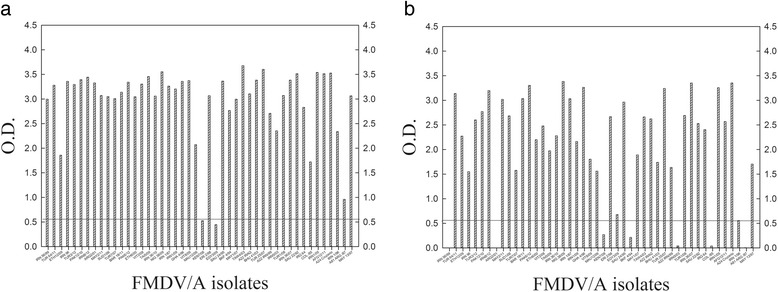
Reactivity of the two mAbs against different FMDV/A isolates in a DAS ELISA. A rabbit antiserum pool to different topotypes of FMDV/A was coated onto microtitre plates. Thereafter, FMDV/A isolates were added to the plates and detected with the mAb #5 (**a**) and #7 (**b**). The lines at the OD value of 0.55 are indicated

The reactivity of the two mAbs to different field isolates of FMDV/A was examined. The mAb #5 reacted with all 46 field isolates archived at the National Centre for Foreign Animal Disease (NCFAD) indicating the binding epitope of mAb #5 is conserved (Fig. 1a). In contrast, mAb #7 failed to react with five of 46 FMDV/A isolates (A/IRN56/99, A/ARG2/01, A/IRN5/03, A/COL/85 and A/ARG/87) (Fig. 1b). Relatively low binding affinity (with optical density (OD) < 0.55) was observed for mAb #5 with two isolates and for #7 with two isolates (Fig. 1). To characterize the mAb–binding sites, their reactivity against native and denatured FMDV/A was examined using an indirect ELISA. Both mAbs failed to react with the denatured FMDV/A, indicating that their binding sites were conformational (data not shown).

### Identification of mAb–binding sites using mAb resistant mutant selection and sequencing

The mAb resistant mutant selection technique was used to identify the binding sites of the two mAbs on the surface of viral particles. The reactivity of the two mAbs to the matching mutants demonstrated a gradual decline, until it was undetectable at passage six, indicating that the mAb binding sites were fully depleted in the selected mutants (data not shown).

The sequences of P1 region that encodes capsid proteins (VP1, VP2, VP3 and VP4) were determined for two mAb escape mutants, and compared with that of parental FMDV/A22 Iraq.

**Fig. 2:**
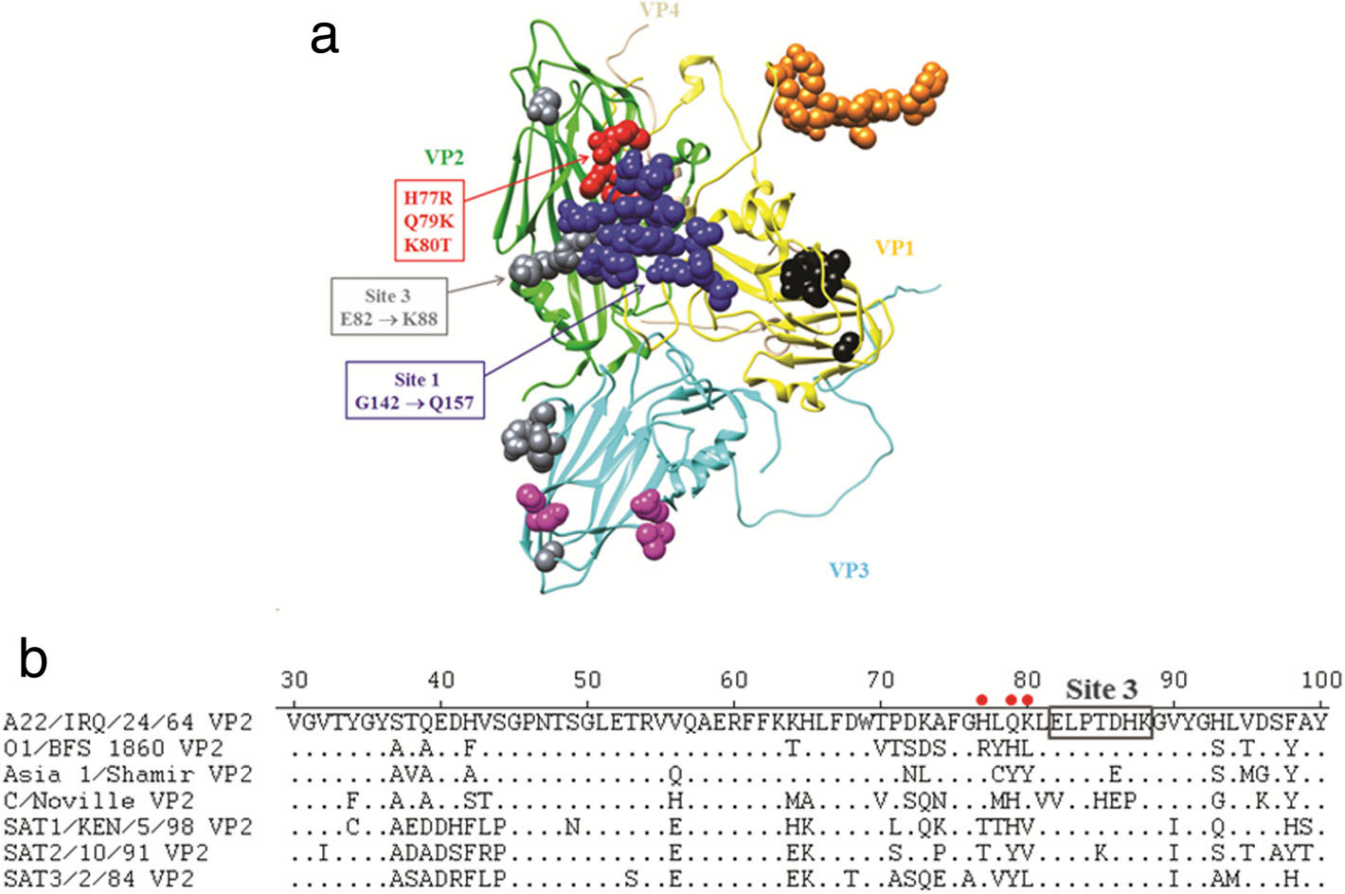
Antigenic sites identified in the capsid crystal structure of the FMDV/O1 BFS 1860. **a** The O1 BFS 1860 asymmetric unit (PDB # 1FOD) was manipulated with Chimera, consisting of 1 VP1 (*yellow*), 1 VP2 (*green*), 1 VP3 (*cyan*) and 1 VP4 (*tan*), with previously identified antigenic sites shown as blue spheres (*site 1*), *orange spheres* (*site 2*), *gray spheres* (*site 3*), *black spheres* (*site 4*) and *magenta spheres* (*site 5*), and with A22 Iraq escape mutant mutations (H77R, Q79K and K80T) depicted as red spheres, respectively. **b** Partial amino acid alignment of the VP2 sequences of representative FMDV serotypes. Positions of the FMDV/A22 Iraq escape mutant mutations are indicated as red dots and the adjacent FMDV/A antigenic site 3 is shown in a box

In both escape mutants, the mutations were observed in VP2. The mutant selected using mAb #5 contained two amino acid substitutions Gln79 to Lys and a Lys80 to Thr, while, the mutant selected using mAb #7 had amino acid substitutions His77 to Arg, and Lys80 to Thr. These substitutions are located in the external part of viral particles and are adjacent to antigenic sites 1 (G142 → Q157) and sites 3 (E82 → K88) as previously identified [16, 17] (Fig. 2a). Amino acid sequence alignments of the A22 Iraq VP2 with those of representative viruses from the other 6 serotypes revealed amino acids are highly variable in the region near the antigenic site 3 of FMDV/A (Fig. 2b). The VP2 alignments of all 46 serotype A isolates indicated that amino acid (aa) at position 80 is highly conserved, whilst at position 77, (ETH/6/2000, GHA/4/1996 and IRN/1/1996) had an amino acid substitution of His to Tyr; the isolate (COL/85) had an amino acid change of His to Glu, and another isolate (ARG/87) had a change of His to Asp.

Examination of VP2 sequences of 45 FMDV/A isolates with that of A22 Iraq revealed various unique amino acid substitutions at or near mAb #7 binding sites for the five non-binding isolates to mAb #7. These substitutions are: an Asp → Asn at position 72 for A/IRN/56/1999; Glu → His at position 79 and Lys → His at position 88 for A/ARG/2/2001; Thr → Val at position 85 for A/IRN/5/2003; His → Glu at position 77, Thr → Ser at position 85, and Lys → Asn at position 88 for A/COL/85; His → Asp at position 77 and Lys → His at position 88 for A/ARG/87. All these substitutions are surface-exposed residues when viewed in the 3D structure of FMDV/A.

### Development of the FMDV/A cELISA

A cELISA to detect antibodies against FMDV/A was developed using the two FMDV/A specific mAb mixture and optimized with a serum of known titres. The BEI inactivated FMDV/A24 Cruziero was used as antigen in the cELISA, since there was no significant difference as compared to A22 Iraq (data not shown).

**Fig. 3:**
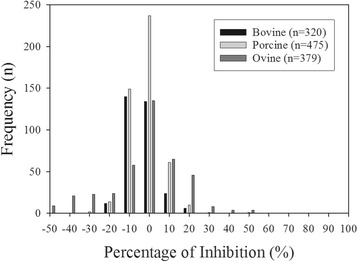
Frequency distribution of the negative sera tested using the FMDV/A cELISA. Serum samples were collected from disease–free bovine (*n* = 320), porcine (*n* = 475) and ovine (*n* = 379) in Canada. Rabbit polyclonal anti–FMDV/A serum pool was coated onto microtitre plates, thereafter BEI inactivated FMDV/A antigen was added. Equal volumes of diluted sera and mAbs (#5 and #7) were added to the plates and allowed to compete with antibodies in serum samples. Results are expressed as the percentage of inhibition

To determine the assay specificity, a total of 1,174 negative sera were tested. The frequencies of the percentage of inhibition (% inhibition) generated from these sera had a normal distribution (mean −1.14%, standard deviation 14.15; Fig. 3). The cut–off value was determined as <50% inhibition [18]. Three of the samples exceeded this cut–off value, which produced a diagnostic specificity of 99.7%.

### Evaluation of the FMDV/A cELISA using sera from experimentally infected animals

**Fig. 4:**
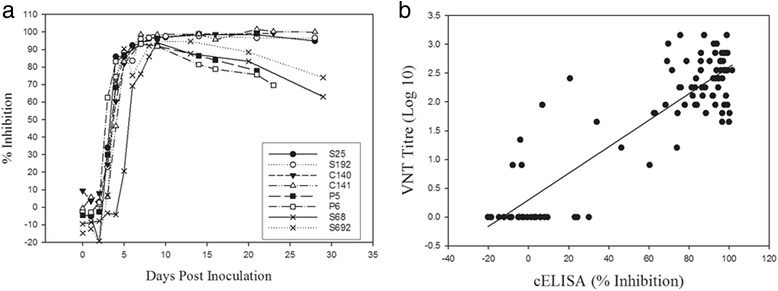
Comparison of FMDV**/**A cELISA and VNT using sera from FMDV/A experimentally infected animals. “S”, “C” and “P” samples correspond to sera from sheep, cattle and pigs. The samples, S68 and S692 were infected with A22 Iraq, others with A24 Cruzeiro. Sera were collected daily up to 10 days post infection (dpi), and then at 7 days intervals after 10 dpi. **a** Serial blood samples were diluted (1:10) and tested using FMDV/A cELISA, and (**b**) scatter plot representing the percentage of inhibition in cELISA versus VNT titres for 99 serial blood samples from eight animals. The line is fitted by linear regression (*r* ^2^ = 0.78, *p* < 0.0001)

The antibody responses to FMDV/A in sera from experimentally infected cattle, sheep and pigs (A24 Cruzeiro or A22 Iraq) collected at various days post inoculation (dpi) were examined using the FMDV/A cELISA. Antibody responses were negative from 0 to 4 dpi (Fig. 4a). At 5 dpi all serum samples were positive for antibodies to FMDV/A, and remained positive until the end of the experiments (21–28 dpi). The FMDV/A cELISA were compared with the gold standard serological test VNT using 99 serial blood samples from eight animals. The Cohen’s Kappa is 0.977 with 95% CI of 0.93–1.0, which indicates a strong agreement (94%) between the cELISA and VNT (Fig. 4b).

**Fig. 5:**
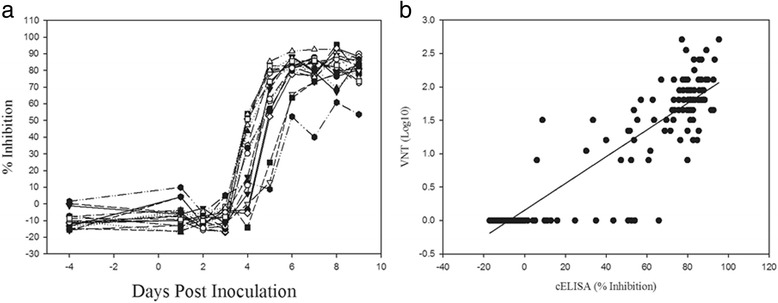
Comparison of A/cELISA and VNT using sera from FMDV/A Vietnam/15/2012 infected sheep. Sheep (*n* = 18) were coronary band infected. Sera were collected daily during the course of experiments. **a** Serial blood samples were diluted (1:10) and tested using FMDV/A cELISA, and (**b**) scatter plot representing the percentage of inhibition in cELISA versus VNT titres for180 serial blood samples from eighteen animals. The line is fitted by linear regression (*r* ^2^ = 0.81, *p* < 0.0001)

Sera from eighteen sheep infected with FMDV/A Vietnam/15/12 were tested to evaluate the ability of the FMDV/A cELISA to detected antibodies against a contemporary isolate. Antibodies were detected in 83.3% of the sheep at 5 dpi and 100% at 6 dpi (Fig. 5a). The calculated diagnostic sensitivity of A/cELISA was 99.3% (98.5–100%). Similar results were observed for VNT. A scatter plot of cELISA% inhibition against VNT titres for 180 serial blood samples from 18 animals is shown in Fig. 5b. The Cohen’s Kappa is 0.90 with 95% CI of 0.71–0.89, which indicates a strong agreement (90%) between the cELISA and VNT. However, the correlations between cELISA and VNT for a few samples collected early after infection (4–6 dpi) were not as good as samples collected at other times.

To determine cross–reactivity, the FMDV/O positive sera from sheep vaccinated and challenged with FMDV/O [19] were tested using the FMDV/A cELISA. The results showed no cross–reactivity up to 35 days post challenge (data not shown). However, there was cross–reactivity (25–30%) with sera from experimental inoculated animals using FMDV serotype O, Asia1, C, SAT 2 and SAT 3.

## Discussion

The purpose of this study was to produce FMDV/A–specific mAbs and use them to develop a cELISA for the detection of FMDV/A antibody. A panel of FMDV/A specific mAbs was successfully generated, two of which competed with a polyclonal serum from FMDV/A infected cattle in a cELISA. The binding sites of the two mAbs were determined to be conformational epitopes exposed on the surface of VP2, located at amino acid positions 77–80, adjacent to antigenic sites 1 (G142 → Q157) and 3 [16, 17]. These sites are known to be immunologically important for FMDV serotype O, A and C [17, 20, 21]. In addition, it has been shown that amino acids 70–80 of VP2 are located in close proximity to the VP1 G–H loop [22]. Mutations within these sites can affect the antigenicity of FMDV. These results suggest that variations at amino acids 70–80 of VP2 domains could be important to the antigenic diversity of FMDV in addition to the hyper–variable VP1 G–H loop (aa 140–160). Being an immune–dominant site increases the chances that a serological assay based on this region will be sufficiently sensitive.

A FMDV/A cELISA using a mAb produced with a VP1 peptide corresponding to the G–H loop was reported previously [14]. However, evaluation of the reactivity of the mAb was limited to two FMDV/A isolates in that study. Therefore, the reactivity of the mAb to other isolates including recent outbreak isolates is not known. Considering that VP1 is the most variable FMDV structural protein [23, 24], it is highly likely that recent isolates of FMDV/A might not react with mAbs produced with VP1 of older isolates. In contrast, there is less divergence and variation in FMDV VP2 and VP3 structural proteins [17]. Conservation of the binding epitopes of FMDV mAbs is an important factor in the choice of appropriate mAbs for diagnostic assays. Therefore, the fact that the two mAbs reacted to the antigenically dominant VP2 of FMDV renders them ideal for a FMDV/A cELISA. The mAb #5 recognized a highly conserved epitope and reacted to 46 FMDV/A isolates, including recent outbreak isolates from 2007 to 2012.

The mAb #7 failed to react with five of the 46 FMDV/A isolates. Examination of VP2 sequences of 45 FMDV/A isolates with that of A22 Iraq revealed various unique amino acid substitutions at or near mAb #7 binding sites for the five non-binding isolates to mAb #7. All substitutions are surface-exposed residues when viewed in the 3D structure of FMDV/A. Thus, it is likely that amino acid alterations may affect binding of mAb # 7 to these five isolates.

There were no observed amino acid substitutions at the mAb binding sites for the low–binding isolates. A possible explanation for the poor binding may be that other mutations occurred that changed the conformational structure of the mAb binding sites, thus affecting the mAbs’ binding.

Since FMDV/A is the most antigenically diverse of the seven serotypes [5], the pair of monoclonal antibodies reacting to different antigen sites is necessary to increase the chance of detecting of antibodies to all strains of FMDV/A, and reduce cross–reactivity with other FMDV serotypes.

The FMDV/A cELISA consistently detected antibodies to FMDV/A in FMDV/A infected animal of different species and was comparable to the gold standard VNT for antibody detection. The Cohen’s Kappa was 0.80–0.977 with strong agreements (90–94%) between the cELISA and VNT indicating the potential use of the cELISA instead of VNT for large screening. However the correlations between cELISA and VNT for a few samples collected early after infection (4–6 dpi) were not as good as samples collected at other times. VNTs detect neutralizing antibody activity and ELISA may detect different antibody subsets. The poor correlation may because of individual variation after FMDV inoculation. Vratskikh et al. [25] provide evidence for extensive differences in the specificities and relative proportions of antibody populations induced by yellow fever vaccination in different individuals. Furthermore, substantial variation was found in the ratio between virion ELISA reactivities and neutralization titers, suggesting a strong influence of antibody subset composition of individual sera. Unlike the VNT, cELISA does not provide antibody titres which are important for post–vaccine monitoring.

The diagnostic specificity of this FMDV/A cELISA was 99.7%, which was comparable to a previously reported FMDV/A cELISA [14]. There was no cross–reactivity when testing sera from FMDV/O1 Manisa vaccinated/challenged sheep in the FMDV/A cELISA. However, there was cross–reactivity with sera from experimental inoculated animals using FMDV serotype O, Asia1, C, SAT 2 and SAT 3. Similar to previous finding where cross–reactions were observed using a FMDV/O cELISA when testing sera collected from animals vaccinated and challenged with FMDV A, C and Asia1 [26]. A blocking ELISA for FMDV/A was found to cross–react strongly with serum positive for antibodies to FMDV/Asia 1 [14]. Hedger et al. [27] found that animals undergoing natural infection with one type of FMDV (type O) had equal or even higher serum antibody titres against one or more of the other types tested (types A, C, Asia 1 and SAT 1). Thus, it remains unclear whether the cross-reactivity among serotypes is caused by low assay specificity, or heterotypic humoral responses following virus infections [28]. Though cross–reactivity among FMDV serotypes is undesirable, it is preferable when sensitivity is the aim. When testing vaccinated populations, these cross-reactions are not of great concern since the serotype of the vaccine strain should be known.

## Conclusion

A panel of FMDV/A specific mAbs was generated and two mAbs specific for antigenically dominant sites located on the structural proteins VP2 were used in the development of a simple and reliable FMDV/A cELISA for the detection of FMDV/A–specific antibodies. The major improvement this FMDV/A cELISA offers over similar assays published previously [14] is the use of mAbs that react with VP2 which is more conserved than VP1.

## Methods

### Virus preparation

All FMDV viruses used in this study were provided by the FAO/OIE World Reference Laboratory for FMD (Pirbright Institute, Pirbright, UK) and listed in Additional file 1: Table S1. The viruses were amplified using either Baby hamster kidney–21 (BHK) or Mengeling–Vaughn porcine kidney (MVPK) cells [29] cultured in Alpha Modification of Eagle’s medium (AMEM; WISENT Inc. Canada). Culture supernatants were harvested and clarified upon observation of complete cytopathic effect (CPE). For immunization, virus was inactivated with 2–Bromoethylamine Hydrobromide (BEI, Sigma-Aldrich, USA) and purified as previously described [8].

### Generation and purification of monoclonal antibodies

Mouse inoculation and hybridoma production were performed as previously described [8]. Briefly, mice were immunized using BEI inactivated FMDV/A22 Iraq three times at 4 week intervals. Spleen cells from immunized mice were then fused with myeloma cells (P3X63 Ag8.653, ATCC, Rockville, MD, USA). Hybridoma supernatants were selected using a FMDV/A double antibody sandwich (DAS) ELISA. FMDV/A antibody positive clones were subcloned, the isotype determined using a mouse monoclonal antibody isotyping kit (Roche, Indianapolis, IN, USA) and the antibodies purified as previously described [30].

### ELISAs

DAS ELISAs for hybridoma screening, mAbs’ specificity and cross–reactivity testing were performed as previously described [15].

An indirect ELISA was used to characterize the mAbs’ binding epitopes. The ELISA and virus denaturation was performed as previously described [15]. Briefly, microtitre plates (Nunc Maxisorp®, ThermoFisher Sientific, Rochester, NY, USA) were coated with purified and denatured FMDV/A diluted in 0.1 M bicarbonate, pH 8.3 and incubated overnight at 4 °C. The plates were blocked with 5% skim milk in a washing buffer (0.05% Tween20 in 0.01 M phosphate–buffered saline (PBS–T)) at 37 °C. After incubation, hybridoma culture supernatants were added to the plates. Then a horse–radish peroxidase (HRP)–conjugated goat anti–mouse IgG (Jackson ImmunoResearch Laboratories Inc., West Grove, PA, USA) was added. For detection, 2, 2′–Azino diethylbenzothiazoline sulfonic acid substrate (ABTS, Roche) was added and the OD at 405 nm was measured using a plate reader (Photometer Multiskan Reader, Labsystems). Each incubation step was 1 h at 37 °C and followed by washing three times with the washing buffer.

### Isolation of mAb–resistant mutants

FMDV/A22 Iraq (10^7^ TCID_50_/ml) was mixed with purified mAbs #5 and #7 for 30min at 37 °C. The virus/mAb mixture and virus only (control) were added onto MVPK cells in T25 flasks. Each flasks were incubated at 37 °C until 100% CPE was observed. The culture supernatants were collected and clarified by centrifugation at 200 × g for 5min. The collected virus was used for the next passage. The procedure was repeated six times. The two mutants were plaque–purified and analyzed [30].

### Sequencing of the P1 region of mutant and parental FMDV/A22 Iraq

The sequence was analyzed as previously described [31]. Briefly, genomic RNA was extracted from parental FMDV/A22 Iraq and its mutants. Available whole genome sequences of FMDV/A from GenBank were aligned. Terminal oligonucleotide primers complementary to the L gene (5′– TGYGTYACCTCYRAYGGKTGGT –3′) (Invitrogen, Carlsbad, CA, USA) and 2B gene (5′– GAAGAAGAARGGYCCRGGGTTGG –3′) were designed for reverse transcription polymerase chain reaction (RT–PCR). Additional primers for sequencing the whole P1 region were chosen from the most conserved region of the FMDV/A sequence alignments (i.e. 5′–CAACACHCARAACAAYGAYTGGTT–3′, 5′–GCNTAYATGAGRAAYGGYTGGGA–3′, 5′–TGACCCYGYYTAYGGYAWGGTG–3′, 5′–CAACMAAYGTRCAGGGVTGGGT–3′, 5′–TACACYGCRCCRCACCG–3′, 5′–ATYTCMARRTCDGARAAGTAGTA–3′, 5′–CACCATGTAVCGGGCYTTTGA–3′, 5′–ACCATBACVACCARYGTCCA–3′ and 5′–CCAAADGSYTTRTCMGKTGTCCA–3′). Full–length cDNA of the P1 regions of each virus were synthesized from genomic RNA followed by further PCR amplification of the cDNAs. Sequencing was performed in both directions using an ABI Prism BigDye Terminator v3.1 Cycle Sequencing Ready Reaction kit (Applied Biosystems, Carlsbad, California, USA) and an Applied Biosystems Genetic Analyzer DNA Model 3130X. Pairwise nucleotide sequence alignments were performed using the Martinez–NW method [32] and the Lipman–Pearson method for protein alignments [33].

### Identification of the monoclonal antibody binding sites on the 3D structural model of VP2

Amino acid sequences of the mutant FMDV/A22 Iraq VP2s were aligned with those of FMDV/O1 BFS 1860 VP2 to identify relative locations of the FMDV/A22 Iraq mutations in the crystal structure of the FMDV/O1 BFS 1860 (PDB # 1FOD; [34]). A molecular graphics image was produced using the UCSF Chimera package from the Resource for Biocomputing, Visualization and Informatics at the University of California, San Francisco [35] and the resulting images imported into Adobe Photoshop for editing.

### Development of a cELISA for detection of serum FMDV/A antibodies using the FMDV/A monoclonal antibodies

The two mAbs (#5 and #7) and BEI inactivated FMDV/A antigen were used in the development of an FMDV/A cELISA. Optimal antigen concentration and antibody dilutions were determined by checkerboard titrations using a serum raised against A24 Cruzeito. Briefly, microtitre plates were coated overnight at 4 °C with 100 μl/well of an antiserum which was pooled from rabbits infected with either one of the following FMDV/A isolates: A24 Cruzeito, A22 Iraq, Iran 1/96 or Col/85. Plates were washed five times with washing buffer, followed by the addition of 100 μl of BEI inactivated FMDV/A24 Cruziero diluted in buffer (2% normal bovine serum and 2% normal rabbit serum in PBS–T) and incubated at 37 °C for 1 h with agitation. After washing, 50 μl of heat–inactivated test sera (final 1:10 dilution) in duplicate, and an equal volume of the mixture of the two diluted mAbs were added to specific wells with the final concentrations 9 ng/well and 35 ng/well for mAb #5 and #7 respectively. Plates incubated for 1 h at 37 °C. After further washing, 100 μl of HRP–conjugated goat anti–mouse IgG (1:2000) was added and incubated for 1 h at 37 °C with agitation. Plates were washed again and 3,3′,5;–tetramethyl benzidine dihydrochloride substrate (TMB, Sigma–Aldrich, St Lucia, MO, USA) added for colour development. The reaction was stopped with the addition of 50 μl/well of 2 M sulfuric acid after 15min incubation at room temperature. The OD was determined at 450 nm using a plate reader. For each washing step, the plates were washed five times with the washing buffer.

Results were calculated based on strong positive (Q1) and negative reference sera (Q3) using the following formula: % inhibition = [(Q3 OD – test sample OD)/(Q3 OD – Q1 OD)] × 100%.

### Negative and positive sera

Negative serum samples were collected from naïve cattle (*n* = 320), pigs (*n* = 475) and sheep (*n* = 379) in Canada, a FMD free–country. The positive sera (A24 Cruzeio and A22 Iraq) were obtained from animals (cattle = 2, pigs = 2 and sheep = 4) experimentally infected with FMDV [10]. Additional sheep (*n* = 18) were infected with FMDV/A Vietnam/15/2012 via the coronary band. Serum samples were collected daily.

### Virus neutralization test (VNT)

The VNT was performed with FMDV A22 Iraq and IBRS2 cells as described previously with modifications [7, 36]. Serum samples were heat inactivated (56 °C, 30min) before neutralization assays. Sera with titres >1.5 log_10_ (1:32) were considered positive [7].

### Data analysis

Cohen’s kappa coefficients were calculated to evaluate interrater agreement between cELISA and VNT using SAS.

## Additional file

Additional file 1: Table S1. Viruses used in this study. (DOCX 17 kb)
