# Supplementary material for: Generation of mAbs to foot–and–mouth disease virus serotype A and application in a competitive ELISA for serodiagnosis
Source: Virol J. 2016 Nov 28;13:195. doi: 10.1186/s12985-016-0650-z (PMC5126828; doi:10.1186/s12985-016-0650-z)
Supplement: Additional file 1: Table S1. — Viruses used in this study. (DOCX 17 kb) [file 12985_2016_650_MOESM1_ESM.docx]

**Additional file 1**

**Table S1: Viruses used in this study**

FMDV

| **Serotype** | **Strain** | **Topotype** |
| --- | --- | --- |
| A | A22 IRAQ | ASIA |
| A | A22 IRN/99 | ASIA |
| A | AFG12/11 | ASIA |
| A | BAR 18/11 | ASIA |
| A | BHU 41/03 | ASIA |
| A | BKF 4/94 | ASIA |
| A | COL /85 | ASIA |
| A | IRN 1/87 | ASIA |
| A | IRN 1/96 | ASIA |
| A | IRN 36/07 | ASIA |
| A | IRN 36/10 | ASIA |
| A | IRN 56/99 | ASIA |
| A | IRN 96 | ASIA |
| A | IRN1/05 | ASIA |
| A | IRN1/09 | ASIA |
| A | IRN5/03 | ASIA |
| A | IRN8/12 | ASIA |
| A | IRQ 64 | ASIA |
| A | MAU12/06 | ASIA |
| A | MAY 13/97 | ASIA |
| A | MAY1/07 | ASIA |
| A | MAY2/11 | ASIA |
| A | PAK12/10 | ASIA |
| A | PAK6/12 | ASIA |
| A | SAU 22/92 | ASIA |
| A | SAU 24/95 | ASIA |
| A | SUD1/06 | ASIA |
| A | TAI5/09 | ASIA |
| A | TAW4/03 | ASIA |
| A | TUR 25/07 | ASIA |
| A | TUR 64/11 | ASIA |
| A | TUR1/08 | ASIA |
| A | TUR3/12 | ASIA |
| A | TUR7/07 | ASIA |
| A | VIT2/08 | ASIA |
| A | VIT8/09 | ASIA |
| A | VIT/15/12 | ASIA |
| A | EGY3/09 | AFRICA |
| A | ERI 2/98 | AFRICA |
| A | ETH12/09 | AFRICA |
| A | ETH6/00 | AFRICA |
| A | GHA 4/96 | AFRICA |
| A | KEN7/08 | AFRICA |
| A | NIG 38/09 | AFRICA |
| A | A24 Cruzerio/Br/55 | EURO-SA |
| A | A81 ARG /87 | EURO-SA |
| A | ARG2/01 | EURO-SA |
| O | BFS 1860 | EURO-SA |
| C | NOVILLE/SW/1/65 | EURO-SA |
| ASIA 1 | SHARMIR/89 | Group III |
| SAT 1 | KEN 4/98 | NWZ |
| SAT 2 | ZIM 10/91 | Topotype 1 |
| SAT 3 | ZIM 4/81 | Topotype I (SEZ) |

**Other vesicular disease viruses**

| Vesicular stomatitis virus | New Jersey Ogden/topotype Concan |
| --- | --- |
|  | Indiana 1 |
| Swine vesicular disease virus | UK 27/72 |
